# Arthroscopic modified Broström operation versus open reconstruction with local periosteal flap in chronic ankle instability

**DOI:** 10.1007/s00402-021-03949-2

**Published:** 2021-05-16

**Authors:** Moritz Mederake, Ulf Krister Hofmann, Ingmar Ipach

**Affiliations:** 1 Department of Orthopaedic Surgery, University Hospital Tübingen, Hoppe Seyler–Str. 3, 72076 Tübingen, Germany; 2 Orthopädie Straubing, Stadtgraben 1, 94315 Straubing, Germany

**Keywords:** Ankle, Ankle injuries, Ankle lateral ligaments, Chronic lateral ankle instability, All-inside arthroscopic modified Brostrom operation, Fibula periosteal flap

## Abstract

**Introduction:**

The modified Broström operation (MBO) has found widespread use in the therapy of lateral chronic ankle instability (CAI). However, alternative surgical techniques like the open reconstruction using a periosteal flap (RPF) are still an important part of the surgical treatment of lateral CAI. Both procedures differ in terms of the reconstruction material used and the surgical procedure. Comparative studies on the surgical therapy of CAI are limited and generally refer to similar surgical procedures. Aim of this study was to compare the arthroscopic MBO and the RPF.

**Materials and methods:**

We retrospectively analysed 25 patients with lateral CAI after a tear of the anterior talofibular ligament (ATFL). 14 patients received arthroscopic MBO and 11 patients received RPF. We compared the postoperative outcome between both groups with respect to subjective instability, the number of ankle sprains, pain, complications and follow-up operations as well as the American Orthopaedic Foot and Ankle Society (AOFAS) ankle-hindfoot score.

**Results:**

Both surgical procedures resulted in a significant improvement in pain, in subjective instability, in the reduction in the frequency of ankle sprains and improvement in the AOFAS ankle-hindfoot score one year postoperatively. Three months postoperatively, the values for pain and instability of the MBO group were significantly better compared to the RPF. One year after the operation, these differences were evened out. Also in terms of complications and follow-up operations, no significant difference was found between the two procedures.

**Conclusions:**

Both surgical procedures give very good results one year postoperatively in terms of pain, instability, function and complication rate. With significantly better results regarding pain and instability three months postoperatively, the MBO allows a faster recovery in patients operated with this technique.

## Introduction

Ligament lesions, especially ankle sprains are one of the most common injuries in sports and daily activities. A recent meta-analysis from Doherty et al. described ankle sprain incidences of 13.6 in women and 6.94 in men per 1000 exposures [11]. In 80% of all patients just the anterior talofibular ligament (ATFL) is torn, while in 20% of these patients both the ATFL and calcaneofibular ligament (CFL) are affected [7, 12]. Usually, a conservative treatment with immobilization with a cast for some days to reduce swelling, and functional full weight-bearing with an ankle orthosis for 5 weeks with physical exercises leads to comparable or better clinical result as an operative treatment [14, 24, 26]. In severe cases with ligament tear, however, secondary chronic ankle instability (CAI) with repeated ankle sprains can persist in 10 to 30% [14, 23, 30]. A high co-incidence with soft-tissue impingement, anterior bony impingement and cartilage damage has been reported in patients with CAI [22, 24].

A good clinical result without pain or compromised range of motion can only be achieved if the function of the original ligaments is somehow restored.

In 1966 Broström et al. already described a surgical technique for the treatment of CAI with a direct augmentation and gathering of the torn ligaments [8]. This technique has been modified by Gould using the retinaculum extensorum inferius for the reconstruction of the ATFL [28]. Recent advances in the field of minimally invasive techniques may today allow a faster recovery compared to open techniques [32]. The modified Broström operation (MBO) can be performed both openly and arthroscopically [6, 17]. In clinical studies, the arthroscopic and the open technique of the MBO did neither differ in clinical nor radiological outcome [34, 35].

Although the MBO seems to be becoming the standard procedure for this condition, there are also alternative techniques to successfully address lateral CAI. In 1997 Rudert et al. treated 94 patients with lateral CAI performing reconstruction with a local periosteal flap (RPF) achieving good or excellent results. This periosteal technique allows anatomical reconstruction without sacrificing other ligaments or tendons [27]. To the best of our knowledge, no comparison between the MBO and the alternative procedure of the RPF is available in the literature.

The aim of the present study was to compare the outcome after arthroscopic MBO and the open RPF regarding instability, pain, postoperative ankle sprains, and function.

We hypothesized that both methods show comparable results in terms of stability and function. Due to the minimally invasive procedure of the arthroscopic MBO, we expected a faster recovery of the patients treated with this procedure.

## Material and methods

We performed a retrospective study comparing the two different operative techniques explained below. All patients received an operative treatment with either a reconstruction with arthroscopic MBO or RPF. Patients were treated in the Department of Orthopaedic Surgery of the University Hospital of Tübingen and in the Praxisklinik Straubing.

Since the study was designed retrospectively, the patients had no choice which surgical method to receive. All operations were carried out by experienced senior physicians.

### Participants

Inclusion criterion was a performed stabilization operation of the lateral ligaments with either the MBO or the RPF. Diagnosis and indication for surgery were based on medical history, clinical examination and further radiographic imaging including conventional X-ray in 2 planes and MRI imaging from each patient. Indication for surgery was a lateral CAI with repeated ankle sprains and failure of conservative treatment, e.g. physiotherapy or braces. The minimum duration of failed conservative treatment before considering surgery was 3 months. Exclusion criteria were a medical history with a chronic pain disorder, therapeutic anticoagulation and unsteady gait caused, for example, by underlying neurological diseases.

### Surgical technique of arthroscopic MBO

**Fig. 1:**
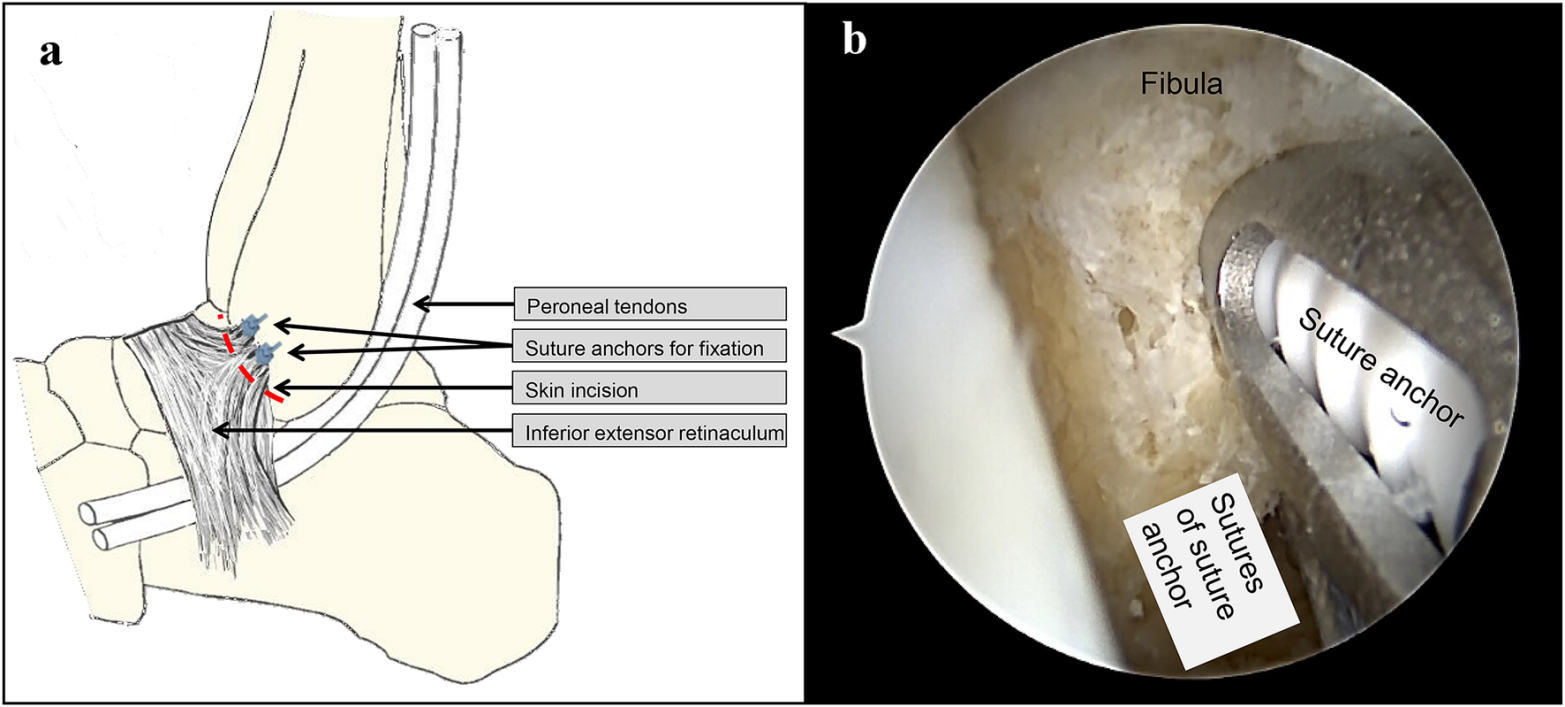
**a** Diagram of the modified Broström operation: The lateral stabilization is achieved by sewing the inferior extensor retinaculum to the periosteum of the lateral malleolus, where it is fixated by means of two suture anchors (modified after Bell SJ, Walthour CS, Provencher MT, Sittler DF. Chronic Lateral Ankle Instability: The Broström Procedure. Operative Techniques in Sports Medicine. 2005). **b** Arthroscopic view with prepared tip of the fibula. The first suture anchor is fixed and the second one is now also inserted. The next step is to pass the sutures through thus sewing the inferior extensor retinaculum to the periosteum of the lateral malleolus

The arthroscopic MBO was performed using the Arthrex Broström Repair implant system (Arthrex Inc. Naples, Florida, USA). A standard anteromedial arthroscopic portal and a secondary anterolateral portal with the protection of neuro-vascular structures are established. Viewing from the anteromedial aspect of the joint a debridement is performed with special attention paid to the preparation of the anterior aspect of the distal fibula with the anatomical origin of the ATFL. Through the anterolateral portal onto the inferior aspect of the fibula a SutureTak drill guide and drill bit are used to create two bone tunnels 5 mm and 10 mm proximal to the tip of the distal fibula. These two drill holes are armed with a 3 mm Biocomposite SutureTak anchor. 15 mm ventrodistally from the tip of the fibula 4 exit points for the suture are marked. Using a Micro SutureLasso, all four sutures are shuttled through the marked points. In the next step, all four sutures are passed subcutaneously through the anterolateral arthroscopic portal. While holding the foot in slight eversion, the sutures are fastened down to the fibula thus sewing the inferior extensor retinaculum to the periosteum of the lateral malleolus (Fig. 1). After surgery, all patients are immobilized in a walking boot for four weeks with the first 14 days with partial load bearing of 10 kg and then full weight bearing for another 14 days. Afterwards full weight bearing is allowed while wearing an ankle orthosis for another 8 weeks. Patients additionally start with physiotherapy with peroneal and proprioceptive exercises at 6 weeks postoperatively. Return to sport is allowed after 3 months.

### Surgical technique of the RPF

**Fig. 2:**
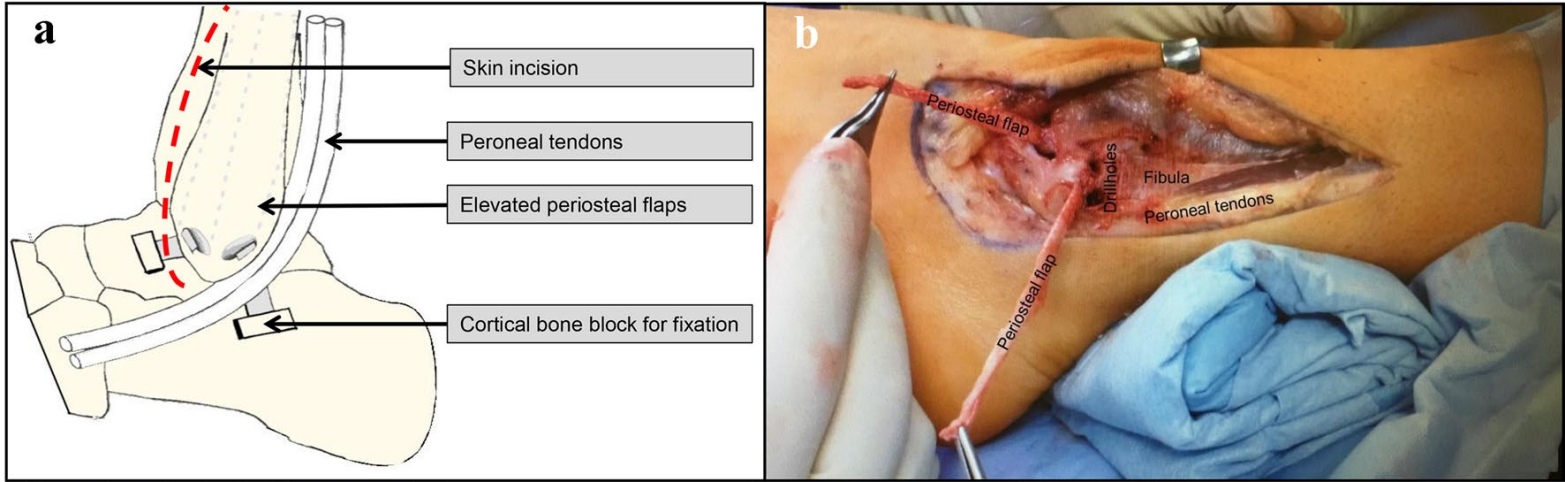
**a** Diagram of the reconstruction with two local periosteal flaps along the anatomical position of the anterior talofibular and talocalcaneal ligaments. Two cortical bone blocks are used for fixation of the flaps by staples (modified after Rudert M, Wulker N, Wirth CJ. Reconstruction of the lateral ligaments of the ankle using a regional periosteal flap. J Bone Joint Surg Br. 1997). **b** Intraoperative situs with prepared periosteal flaps. The drillholes are prepared to pull through the flaps and fixate them at the sites of insertion of the ATFL and CFL (*ATFL* anterior talofibular ligament, *CFL* calcaneofibular ligament)

The skin incision is located anterior to the distal fibula curving in the posterior direction distally at the lateral malleolus. Then two periosteal flaps from the fibula are prepared and elevated from proximal to distal. The next step is to make two drillholes at the tip of the fibula in the direction of the attachments of the ATFL and the CFL. The two periosteal flaps are now pulled through the drillholes. The insertion sites of the ATFL and the CFL have to be exposed and a cortical bone block of 10 × 10 mm is then removed. The dorsal flap is fixed in slight equinus of the foot. It is directed deep to the peroneal tendons and held under tension. The fixation with the cortical block at the prepared site of insertion of the CFL is made by a staple. Fixation of the anterior flap is made in the same way at the prepared site of insertion of the ATFL, but in a neutral position with the hindfoot in valgus at the subtalar joint (Fig. 2).

After surgery, the foot is held in a neutral position with a plaster splint for two weeks. Partial load bearing of 10 kg for 6 weeks postoperatively is allowed. An orthosis is then supplied for another 4 weeks with full weight bearing. Patients additionally perform physiotherapy with peroneal and proprioceptive exercises starting at 6 weeks postoperatively. Return to sport is allowed after 6 months.

### Outcome measurement

Results were evaluated using several questionnaire-based scales. To compare the number of ankle sprains, the patients answered a questionnaire stating the frequency of ankle sprains preoperatively and postoperatively. A selection from the following frequencies was possible: none, yearly, monthly, weekly and daily. These details were used as ordinal values for statistical analysis. With regard to the feeling of instability, the patients could indicate “yes” or “no” in a dichotomous manner for the time points pre- and postoperatively. Pain was recorded with the numeric rating scale (NRS) ranging from 0 with “no pain” to 10 “greatest imaginable pain”. We also used the AOFAS ankle-hindfoot score with a maximum score of 100 evaluating pain (40 points), functional status (50 points) and alignment of the foot with the ground (10 points). The functional dimension of the AOFAS ankle-hindfoot score comprises functional limitation, use of supports, distance covered, ground characteristics, step alterations and sagittal movements. Furthermore, previous operations and follow-up operations were recorded, too.

### Statistical analysis

Statistical analyses were conducted using IBM SPSS Version 20 (IBM, Armonk, NY, USA) and Microsoft Excel (Microsoft, Redmond, WA, USA). Distributions of variables within the groups were assessed by histograms and a non-parametric approach was chosen. Continuous variables are presented as medians and ranges, and categorical variables as frequencies. Comparison between groups was performed by Mann–Whitney U-test, Wilcoxon-test or Chi-Square-test as appropriate. All reported p-values are two-sided, with a significance level of 0.05, and have not been adjusted for multiple testing. Power analyses were conducted using G*Power Version 3.1.9.6.

## Results

The analysed collective consisted of 25 patients (22 women and three men), 14 of whom received an arthroscopic MBO and 11 a RPF. The operations were carried out between 2002 and 2018. Median patient age at the time of operation was 35 years (range 17—54 years). Two patients from the RPF group had previously been operated on in the operating area (both reconstruction of the ATFL, not otherwise specified). There was no statistically significant difference between the two groups for any of the above-mentioned characteristics (Table 1).

**Table 1:** Epidemiologic characteristics of the study collective

	Total	MBO	RPF	*P*-value
N	25	14	11	–
Age in years (median, range)	35 (17 – 54)	36.5 (17 – 53)	34 (17 – 54)	0.373
Sex (f:m)	22:3	12:2	10:1	0.692
Previous operations (%)	2 (8)	0 (0)	2 (18)	0.250

*MBO* modified Broström operation, *RPF* reconstruction using a periosteal flap

Complications occurred in six patients (24%) of the whole collective. In the MBO group, one suture granuloma and one nerve damage were observed (14% complication rate). In the RPF group, three patients had nerve damage and one patient suffered from wound healing disorders (complication rate 36%) (*p* = 0.199). In total, five patients had to undergo revision (three patients with MBO (21%) and two patients with RPF (18%), *p* = 0.840). In the MBO group, the revisions consisted of a cheilectomy, resection of suture material granuloma and neurolysis. Revisions in the RPF group included one scar revision and one implant removal.

**Fig. 3:**
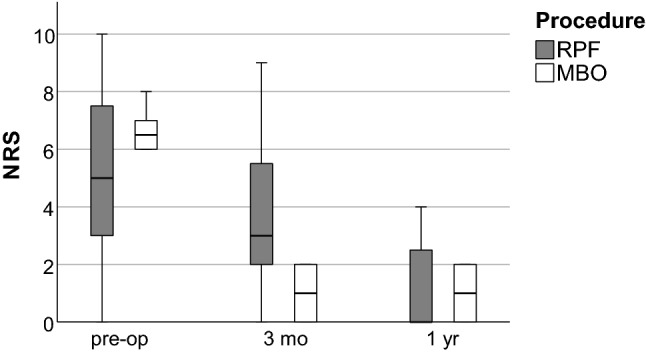
Numeric rating scale (NRS) for pain depending on the time of the examination (RPF: reconstruction using a periosteal flap; MBO: modified Broström operation; pre-op: preoperatively; 3 mo: 3 months postoperatively; 1 yr: 1 year postoperatively)

The median preoperative value on the NRS in the MBO group was 6.5. This value was reduced to 1 three months (*p* < 0.001) and one year (*p* < 0.001) postoperatively. In the RPF group, the median pain was reduced from 5 preoperatively to 3 three months (*p* = 0.261) and 0 one year (*p* = 0.012) postoperatively. Pain reduction was thus significantly greater in the MBO group when compared to the RPF group (*p* = 0.029) at three months postoperatively. One year postoperatively, pain levels were similar in both groups (Fig. 3).

**Fig. 4:**
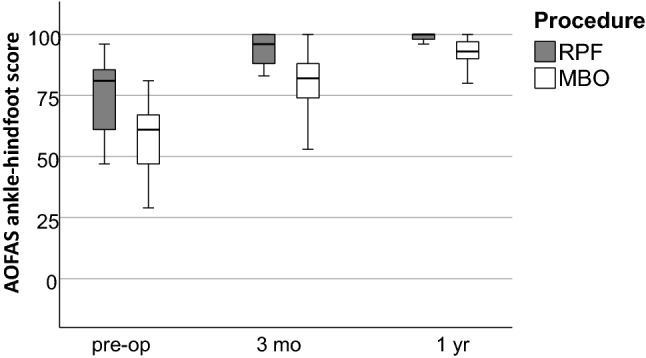
AOFAS ankle-hindfoot score depending on the time of the examination (*RPF* reconstruction using a periosteal flap; *MBO* modified Broström operation; pre-op: preoperatively; 3 mo: 3 months postoperatively; 1 yr: 1 year postoperatively)

To retrospectively assess function, we used the AOFAS ankle-hindfoot score. The patients of the MBO group preoperatively presented with a median score of 61 (range 29—81). A significant improvement to a median score of 82 (range 53 – 100) (*p* = 0.002) at three months and of 93 (range 53 – 100) (*p* = 0.002) at one year postoperatively was documented. The median score of the RPF group was 81 (range 47 – 96) preoperatively. In this group, too, the score improved significantly in the postoperative follow-up with a value of 96 (range 60 – 100) (*p* = 0.004) at three months and a value of 100 (range 71—100) (*p* = 0.01) at one year postoperatively. In direct comparison, the RPF shows significantly better absolute values both preoperatively and at both points in time postoperatively (*p* = 0.011, *p* = 0.013 and *p* = 0.018, respectively) (Fig. 4). However, if the differences between the points in time are compared, no significant differences between the RPF and the MBO emerge (Power 0.35, Cohen ‘s *d* = 0.683).

**Fig. 5:**
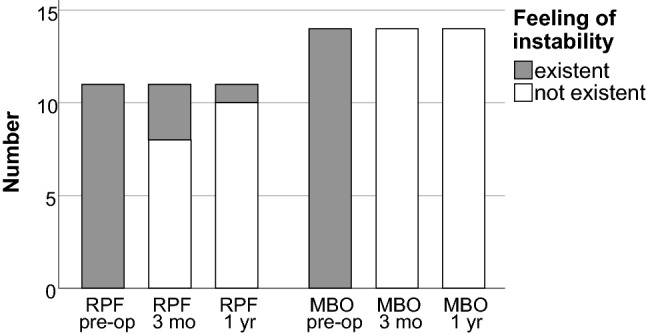
Reported perioperative feeling of instability in both groups (*RPF* reconstruction using a periosteal flap; *MBO* modified Broström operation; pre-op: preoperatively; 3 mo: 3 months postoperatively; 1 yr: 1 year postoperatively)

A feeling of instability was present in all patients preoperatively. In the MBO group, no patient-reported persisting instability postoperatively. In the RPF group, three patients (27%) still reported a feeling of instability at three months postoperatively and one patient (9%) one year postoperatively. In comparison, the MBO group thus showed significantly better stability three months postoperatively (*p* = 0.037) (Fig. 5).

**Fig. 6:**
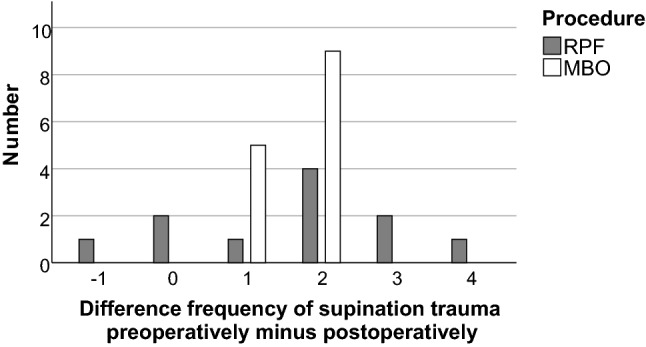
Difference in the frequency of supination trauma from preoperatively to postoperatively. The patients answered a questionnaire stating the frequency of ankle sprains preoperatively and postoperatively. A selection from the following frequencies was possible: none, yearly, monthly, weekly and daily. These details were used as ordinal values from 0 to 4 for calculation of the difference in frequency of supination trauma. Negative values, therefore, indicate an increase and positive values a decrease in the occurrence of supination trauma (*RPF* reconstruction using a periosteal flap; *MBO* modified Broström operation)

Both procedures showed a relevant reduction in supination trauma (Table 2). However, there was no statistically significant difference between both procedures (*p* = 0.065) (Fig. 6).

**Table 2:** Frequency of ankle sprains

		Frequency of ankle sprains preoperatively	Frequency of ankle sprains postoperatively
		N (%)	N (%)
MBO Group	None	0	14 (100)
	Yearly	5 (35.7)	0
	Monthly	9 (64.3)	0
	Weekly	0	0
	Daily	0	0
RPF Group	None	0	6 (54.5)
	Yearly	1 (9.1)	2 (18.2)
	Monthly	7 (63.6)	3 (27.3)
	Weekly	1 (9.1)	0
	Daily	2 (18.2)	0

*RPF* reconstruction using a periosteal flap, *MBO* modified Broström operation

## Discussion

The MBO has found widespread use in the treatment of lateral CAI [3, 6, 17, 19]. It could be shown that the MBO has a good clinical outcome and good subtalar motion with few technical difficulties [8, 9, 19, 34, 35]. In recent years, in addition to the open MBO, the arthroscopic MBO has also increasingly been performed [33]. Both methods have a comparably good therapeutic effectiveness with a similarly low rate of complications. The arthroscopic technique results in a smaller incision but it is more expensive and takes a longer operation time [34, 35]. Biomechanical studies also showed that the two methods do not show any significant differences in terms of torque to failure, degrees to failure, and stiffness [13, 16]. When talking about the MBO, it needs to be pointed out that the ankle lateral ligament repair alone is also still being performed. Interestingly, recent studies show similar good results when comparing a lateral ligament repair alone with a lateral ligament repair with reinforcement by inferior extensor retinaculum [29].

Although the MBO seems to be becoming the standard of care, there are also alternative techniques which are part of the surgical therapy in lateral CAI [21]. One of these frequently performed alternative techniques is the RPF which was suggested by Kuner 1978 [15]. This periosteal technique with reported good or excellent results allows anatomical reconstruction without sacrificing other ligaments or tendons of the foot [27]. Another advantage is the very similar biomechanical properties of the periosteal flap compared to the ATFL [5]. A very good recovery with the restoration of the sporting activity could even be shown for highly active athletes [4]. The RPF has also recently appeared more frequently in studies as an augmentation for the MBO [10]. However, to the best of our knowledge, no comparison between the MBO and the alternative procedure of the RPF is available in the literature. We retrospectively analysed the patient’s outcome for both procedures in terms of pain, stability and function.

One year postoperatively, both groups showed good improvement in function and a significant reduction in pain, subjective instability and the number of ankle sprains with no significant difference between both groups one year postoperatively. However, patients treated with the MBO recovered faster with significantly greater improvement in the feeling of instability (*p* = 0.037) and pain (*p* = 0.029). The arthroscopic MBO, therefore, appears to offer a faster pain reduction than the RPF. Rudert et al. (1997) were able to show for the RPF that pain was postoperatively never present in the majority of patients or only after greater exertion [27]. Even in highly demanding athletes a very good pain situation postoperatively is possible in almost all patients [4]. Unfortunately, there is no comparative study for the RPF that shows the time course of the pain development postoperatively. When looking at the MBO a very similar substantial pain regression from 4–5 (visual analog scale) preoperatively to 1–2 postoperatively has been described as early as 6 weeks postoperatively [10, 34, 35].

Comparing the function of the ankle with the AOFAS ankle-hindfoot score no statistical significant differences in improvement could be demonstrated between the groups. Comparative studies show an improvement in the AOFAS ankle-hindfoot score for the MBO from approx. 60 preoperatively to over 90 postoperatively [34, 35]. With a full return to sports rate of 81% and an improvement in the AOFAS ankle-hindfoot score from 69 to 97, similarly good results have already been achieved in previous studies for the RPF [4, 27]. Therefore, the results available here are similar to comparative studies for function and its improvement [4, 6, 10, 34, 35]. Interestingly, studies comparing the open with the arthroscopic MBO showed comparable good results one year postoperatively, with, however, a faster pain reduction and recovery for the arthroscopic MBO [2, 18, 20].

Although not statistically significant, the complication rate in the RPF group (36%) was more than twice as high as in the MBO group (14%). The most common complications reported in the literature are nerve injuries, wound healing problems and suture material complications, which is consistent with the data of the present study [10, 34, 35]. Because of this risk of entrapment of relevant anatomical structures such as the superficial peroneal nerve, the sural nerve, or the peroneal tendons safe zones have been defined using anatomical studies. These zones should be determined preoperatively [1].

The successful conservative therapy of ankle sprains with only very few patients requiring surgical treatment leads to the problem of small sample sizes in studies looking into surgical strategies of CAI [4, 10, 14, 35]. In patients with ligamentous injuries and persisting ankle instability, however, stabilizing surgery often has to be performed, which is why this topic is of high clinical relevance. The smaller sample sizes also entail the retrospective character of the present study. It is thus possible that over time a certain reporting bias is present in the current data set. Demographic characteristics were, however, comparable between both groups.

The statistical power of certain tests in our study can be classified as small to medium thus being underpowered. This is why results showing no difference could be false negatives in the sense of a type II error. This fact can largely be attributed to the limited sample size. Although the descriptive data for both procedures at one year postoperatively do not suggest a clinically relevant difference, these findings need to be interpreted with caution. Since a large body of research in the CAI field consists of studies with smaller sample sizes, our data may also contribute to future meta-analyses improving our perspective on these procedures.

When interpreting the results of our study it needs to be pointed out that we compared one minimally invasive procedure (MBO) with an open surgical technique (RPF). Although no minimally invasive procedure is available for the RPF it would still have been interesting to also compare the RPF with an open MBO. Looking at other comparative studies of open versus minimally invasive techniques, similar results with less pain and faster recovery for minimally invasive techniques can be seen [20, 31]. It thus appears that parts of the differences observed between the techniques can be attributed to the choice of an open vs. minimally invasive procedure.

One methodical limitation is that the AOFAS ankle-hindfoot score is not recommended by the AOFAS anymore because of several flaws inherent to the questionnaire [25]. As there remains, however, a lack of validated and comparable score alternatives, the AOFAS score keeps being used in various studies dealing with the hindfoot [18, 31, 34].

## Conclusion

The MBO and the RPF are surgical techniques that yield a comparable clinical outcome one year postoperatively. Pain, function, instability and the frequency of ankle sprains improve significantly with both procedures and do not differ significantly from one another. The arthroscopic MBO appears to have the advantage of faster recovery through significantly faster pain reduction and improvement of instability.

From our point of view, RPF can be seen as a good alternative therapy, which can be carried out if the MBO is not technically feasible or available.
